# Hydration Contribution to the Solvation Free Energy of Water-Soluble Polymers

**DOI:** 10.1021/acs.jpcb.5c01009

**Published:** 2025-06-03

**Authors:** Jennifer A. Clark, Jack F. Douglas

**Affiliations:** Materials Science and Engineering Division, Material Measurement Laboratory, 10833 National Institute of Standards and Technology, Gaithersburg, Maryland 20899, United States

## Abstract

We study the solvation free energy of model water-soluble polymers with an emphasis on better understanding the entropic contribution deriving from the formation of a dynamic hydration layer (DHL). To isolate the solvation free energy due to polymer hydration from contributions that arise from changes in the polymer conformation (and thus solvent-accessible surface area) that ordinarily accompany solvation, we restrict a polymer chain in a rod-like configuration. As in recent works, the nanoscale mobility gradient around the polymer chain, defining the DHL, is quantified through the determination of the Debye–Waller parameter, ⟨*u* ^2^⟩, for solvent in the vicinity of the polymer. This gradient enables us to easily visualize the DHL around the polymer. Direct computation of the free energy of solvation indicates a large entropic contribution that correlates with changes in Kirkwood–Buff integrals, which allow us to quantify specific ion effects on polymer solvation. While the water mobility exhibits a significant dependence on the strength of the polymer–solvent interaction in the nanoscale DHL, we unexpectedly found no additional specific ion effect on the mobility within the DHL relative to the bulk solution and, moreover, we find no change in the spatial extent of the DHL to within experimental uncertainty. On the other hand, we find an excess density of CsCl close to the polymer and density depletion of NaCl, consistent with previous suggestions that chaotropic ions partition toward polymer interfaces. Our work indicates that polymer hydration can make a large contribution to polymer solvation free energy, and we expect this phenomenon to be important in relation to understanding the thermodynamics of molecular self-assembly and phase separation processes of water-soluble polymers.

## Graphical abstract

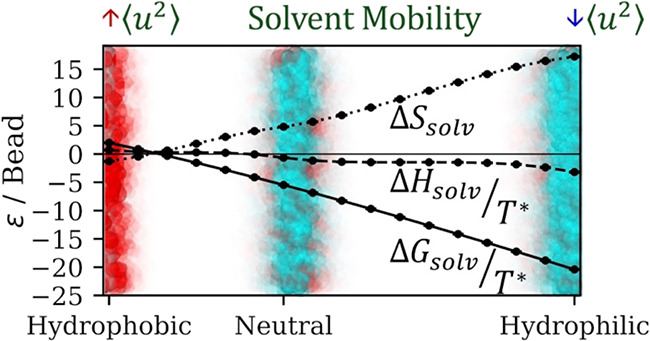

## Introduction

Water-soluble polymers are ubiquitous in many commercial applications ranging from manufacturing, oil recovery, food processing, agricultural applications, personal care products, and diverse medical use cases.^1–5^ The full range of application is large and beyond the scope of this work, but several reviews^1,3^ provide additional information on the subject. Furthermore, water serves as the medium of life and molecular biology: fields that are predominantly characterized by the interactions and phase separation processes of water-soluble polymers.^1,6,7^ As in the case of ordinary polymers, the molecular association and phase behavior of water-soluble polymers is often influenced by changes in temperature, but salts can play an equally important role in phase stability and molecular self-assembly processes.^1,2,4,5,7^ It is well known that the dissolution of polymers in water is often accompanied by a hydration layer around the polymer chain to mediate the effective interpolymer interactions.^1,3,6,8^ We provide a more expansive overview of the relevant literature elsewhere,^9^ and here, we summarize and highlight key concepts relevant to the present work.

In our recent work on the characterization of a *dynamic hydration layer* (DHL) around a zwitterionic polybetaine,^9^ we demonstrated the existence of a layer that has been suggested to greatly influence intermolecular interaction of biomolecules,^10–16^ a phenomenon of potential profound biophysical importance. Such a layer could allow these macromolecules to “sense” each other at relatively large distances and to regulate both their binding^17^ and targeted docking.^18,19^ The existence of a DHL was first made apparent experimentally in terahertz spectroscopy measurements, where the protein concentration was increased until collective effects were observed.^16,20^ The onset of these collective effects are considered to result from the overlap of protein DHLs with a thickness on the order of a nanometer.^16,21,22^ These pioneering terahertz spectroscopy measurements have also been applied to probe the dynamic interfacial layer of a variety of biological molecules.^16,20,23,24^ Other measurement techniques have since been applied to characterize this layer, such as femtosecond-resolved fluorescence,^25^ dynamic neutron scattering,^21,26^ and Overhauser dynamic nuclear polarization,^27–30^ which confirm the same qualitative picture of a DHL having a thickness on the order of a nm, as we found in our atomistic simulations.^9^

Our previous method of characterizing the DHL drew upon ideas developed in the field of glass-forming liquids where the mean square displacement of water molecules on a caging time scale, the Debye–Waller parameter ⟨*u* ^2^⟩, was used to quantify the mobility gradient around the polymer chains. In the present method, we extend our characterization protocol of the hydration layer by calculating the free energy of polymer hydration. In particular, ⟨*u* ^2^⟩ has been utilized in polymer glass-forming liquids to quantify mobility gradients around inorganic nanoparticles in polymer matrices,^31^ and more recently in studies of solvated ions and polymers,^9,32–34^ where it was found to relate to thermodynamic and transport properties.^34–36^ Following previous researchers, we define ⟨*u* ^2^⟩ as the mean-squared displacement (MSD) of the particles on a characteristic caging time scale, *t*_C_, a time scale on the order of a ps in molecular fluids.

Understanding the role of hydration in the thermodynamics of polymers in dilute solution has long been considered but not well understood. Flory–Huggins (FH) theory^37,38^ provides an often useful coarse-grained model of the thermodynamic properties of polymer fluids and solutions, leveraged in polymer physics. Yet, deficiencies in FH theory to describe dilute polymer solution properties were quickly recognized by Flory and Krigbaum^39^ and elaborated on by others in later work, as discussed by Freed.^40^ Nonetheless, the FH model has remained useful for the qualitative insights it offers about polymer solubility and the influence of polymer-excluded volume interactions on polymer solution properties. Importantly from the standpoint of the present work, and as described by Flory and Kringbaum^39^ and its refinement by Yamakawa,^41^ standard FH theory does not address hydration interactions between polymers and the solvent that can lead to large entropic effects to the free energy of mixing. Flory and Kringbaum simply assumed heuristically additive enthalpic and entropic contributions to the mixing free energy parameter, χ, of polymer solutions, which is taken to vary linearly in the polymer volume fraction.^39^ Freed and co-workers^42^ extended the standard FH theory to describe solvation by treating the polymer–solvent interaction as a reversible binding equilibrium between the solvent and polymer and controlled by enthalpic and entropic thermodynamic energetic parameters. This rather complex formalism allowed for the explicit calculation of the entropic contribution from the Flory χ-parameter. The large magnitude of the entropic contribution derived from this formalism is in qualitative accord with experimental studies where it was taken as being purely phenomenological as in the Flory and Kringbaum theory. A shortcoming of this associating solvent theory is that the extended nature of the hydration layer cannot be addressed in this model in its present form, but we may nonetheless expect the prediction of a large entropy of mixing by extended FH theory to be qualitatively valid.

It is apparent from previous studies that the polymer–solvent interaction has an effect on the solvation of the polymer, while this work suggests that there is a significant contribution from the solvent itself. The favorability of the interaction of a solute (in this case, a polymer) with water is often categorized to be either hydrophobic or hydrophilic depending on its free energy of solvation. In this work, we define a polymer to be *hydrophilic*, *neutral*, or *hydrophobic* according to its polymer–solvent interaction energy. In solution, the hydrophobic polymer would collapse, such as at the cloud point in experiments, while the chain dimensions of a hydrophilic polymer would expand and fluctuate, such as a Gaussian chain, depending on the hydrophilicity of the polymer. The perspective of hydrophilicity puts the onus of solvency in water on the solute; however, the framework of polymer physics puts the onus on the solvent as being either *poor* or *good*.^37^ Ultimately, the chain dimensions of the polymer depend on the change in free energy to solvate a polymer in a particular solution, which depends on cohesion between the solute with itself, the solvent with itself, and between each other.

The chain dimensions of a solvated polymer in dilute solution are significantly dependent on temperature, where the response can fall into two categories. One may find it intuitive that a polymer chain would collapse in solution as the temperature is lowered past the lower critical solution temperature (LCST); however, some polymers exhibit an upper critical solution temperature (UCST) as well. LCST behavior is indicative of both a negative enthalpy and entropy of solvation, while UCST behavior is indicative of a positive enthalpy and entropy of solvation.^43^ However, predicting these behaviors is not so simple as the enthalpy and entropy of hydration will both change with temperature.^43^ Such temperature-dependent modifications to theories modeling the contributions have been successful for systems showing LCST behavior.^44–46^ In that case, LCST polymers were discussed to be entropically driven processes, where the entropically favorable mixing is balanced by an entropically unfavorable contraction in density, where a reduction in temperature overturns the favorability.^44,45^ The landscape of such phase behavior can then be tuned with the construction of block copolymers, such as disordered proteins,^47^ containing hydrophilic and hydrophobic moieties. Leveraging the tunable properties of dilute solution polymers or simply understanding the biopolymer behavior requires a better understanding of their thermodynamics.

Consequently, there exists an opportunity cost associated with polymer solvation, although only the enthalpic component is considered in the FH theory (refer to Section S1 of the Supporting Information). Based on our previous research on the DHL of polymers^9^ and ions,^34^ and reviewing the biopolymer literature, we assert that there is also a strong entropic component for the solvent to hydrate a polymer. This research endeavors to validate that hypothesis. However, since the polymer chain dimensions will also be influenced by how well the polymers is solvated, collapsing if in poor solvent and extending if in good solvent,^37^ a study of free polymer chains in solution would involve a difference in solvent-accessible surface area with the contribution of excluded volume interactions.^48^ Such a difference in the surface area would significantly impact the free energy for the various polymer–solvent interaction energies considered in this work. To address this, we constrain the polymer to be an infinitely long chain that cannot fluctuate in solution (or vacuum), thereby eliminating the free energy contributions from polymer–polymer interactions and subsequent effects on polymer–solvent interactions resulting from a change in solvent-accessible surface area. As a result, the free energy changes will be less like a polymer chain fluctuating in solution, and more like systems that can be approximated as rods, such as tobacco viruses^49^ or methyl cellulose.^50^ The simplification of representing polymers as rods in the study of complex phenomena has also been demonstrated theoretically^51^ and in simulation study.^52^ By investigating the solvation free energy of an infinitely long, straight polymer chain, we isolate the enthalpic and entropic effects that a polymer may exert on the solvent, thereby testing whether such an effect exists.

The solvation behavior of polymers in dilute solution is also known to be influenced by salt type and concentration. For uncharged polymers, salts reduce the temperature at which a phase transition occurs.^53^ For polyelectrolytes, salts serve to screen polymer–polymer interactions where charge fluctuations caused by their diffusion between correlated domains and free ions produce a rich effect on these systems.^54^ Considering that we suspect that the solvent too has a rich landscape of behavior around the polymer, the question of how the presence of salt may influence the free energy of solvation remains. We know from our previous work that the mobility of the solvent is depressed for polybetaines,^9^ and we suspect that that depression will have an effect on the free energy of solvation of the polymer. We also know from a previous work that kosmotropic and chaotropic ions similarly depress and enhance solvent mobility, respectively.^34^ We then consider the possibility that the presence of kosmotropic and chaotropic salts would influence our hypothesized solvent contribution to the free energy of solvation. Such an effect would then be relevant to studies relating to the Hofmeister series^55^ such as for proteins and charged polymers.^56^

Up to the present time, the entropic contribution of the DHL on the free energy of polymer hydration has not been characterized. We then examine this contribution to the solvation free energy using molecular dynamics (MD) simulations and then apply Multistate Bennett Acceptance Ratio (MBAR)^57,58^ to determine the free energy of polymer solvation. We demonstrate the free energy change with respect to the solvent–polymer interaction energy parameter, as well as temperature, illustrating the entropic contribution of this layer. To further characterize the effects experienced by solvent in the DHL, that solvent is independently analyzed and compared to the bulk solution, showing alterations in coordination and Kirkwood–Buff integrals. Lastly, although specific ion effects in the free energy are not exhibited due to our simplifying assumption of equal polymer–ion and polymer–solvent interaction energies, specific ion effects shown in the Kirkwood–Buff integrals suggest that effective attraction and repulsion may result from interactions of hydration layers.

## Methods

### Molecular Dynamics Parameters

This work models an infinitely long polymer chain in solutions with or without 1 mol/L NaCl or CsCl in a coarse-grained (CG) MD approach. The simulation boxes were built for each system condition with the MosDef^‡^ suite (i.e., mbuild^‡^ ^59^ and foyer^‡^ ^60^), where we contributed the ability to align a polymer along an axis and attach its termination points (Figure 1). Each simulation box then contains 109,350 CG beads in total where each polymer is 30 beads long and when a concentration of 1 mol/L is relevant, 5,269 salt pairs are present, with the remaining beads defined to be solvent. The polymer beads are attached through a stiff harmonic potential, *U*_Bond_ = *k*_Bond_(*r* – *r*_Bond_)^2^, with a bond length of *r*_Bond_ = 1 σ and a spring constant of *k*_Bond_ = 10,000 ϵσ2 . The box is 30 σ in the *z*-coordinate so that this periodic polymer attaches to itself to restrict its conformation to be linear without further constraints. The CG beads interact through a Lennard-Jones (LJ) potential, where all values of σ and ϵ are equal to unity in LJ units unless otherwise specified. The cations are paired with Cl^–^ as a counterion with an interaction energy of ϵ_Cl,Na/Cs_ = 1. The cations range in their cross-interaction energy parameters with solvent according to their scaled Born radii relative to K^+^ (without fit parameters), as explained in previous work.^61^ This methodology for estimating the total ion–solvent interaction strength for Na^+^ (kosmotropic) and Cs^+^ (chaotropic) results in values of *ϵ*_CS_ = 1.25 and 0.85, respectively, in LJ units with a potential cutoff of *r*_C_ = 3 σ. To represent the charge interaction between the anions and cations, a dielectric constant of either 0.849 or 0.719 was used to scale the charges to produce a Bjerrum length of 1.73 σ at T* = 0.7 or 0.79, respectively, where σ is expected to be 0.4 nm.^61,62^ The characteristic time scale for this LJ system, τ, is on the order of a ps, consistent with the Debye–Waller parameter for water models used in atomistic simulations.^9^ Cross-interactions between the solvent and uncharged polymer are varied to represent hydrophobic and strongly hydrophilic interactions, ϵ_PS_ ∈ {0.4,1.0,2.0}, where for reference, the interaction energy between Li^+^ and solvent for this model is 1.85.^62^ Interactions between salt and the polymer are equal to ϵ_PS_ so that there is no preferential aggregation or repulsion from the polymer due to LJ interactions. A summary of the LJ energy parameters can be found in Table 1.

**Table 1:** Lennard-Jones Interaction Energy Parameters

bead type	P: polymer	S: solvent	A: anion	C: cation
P: polymer	1			
S: solvent	0.4, 1.0, or 2.0	1^a^		
A: anion	ϵ_PA_ = ϵ_PS_	1^a^	1^a^	
C: cation	ϵ_PC_ = ϵ_PS_	Cs^+^: 0.85^a^	1^a^	1^a^
		Na^+^: 1.25^a^		

a Potential parameters are the same as prescribed by Andreev et al.^61,62^

**Figure 1:**
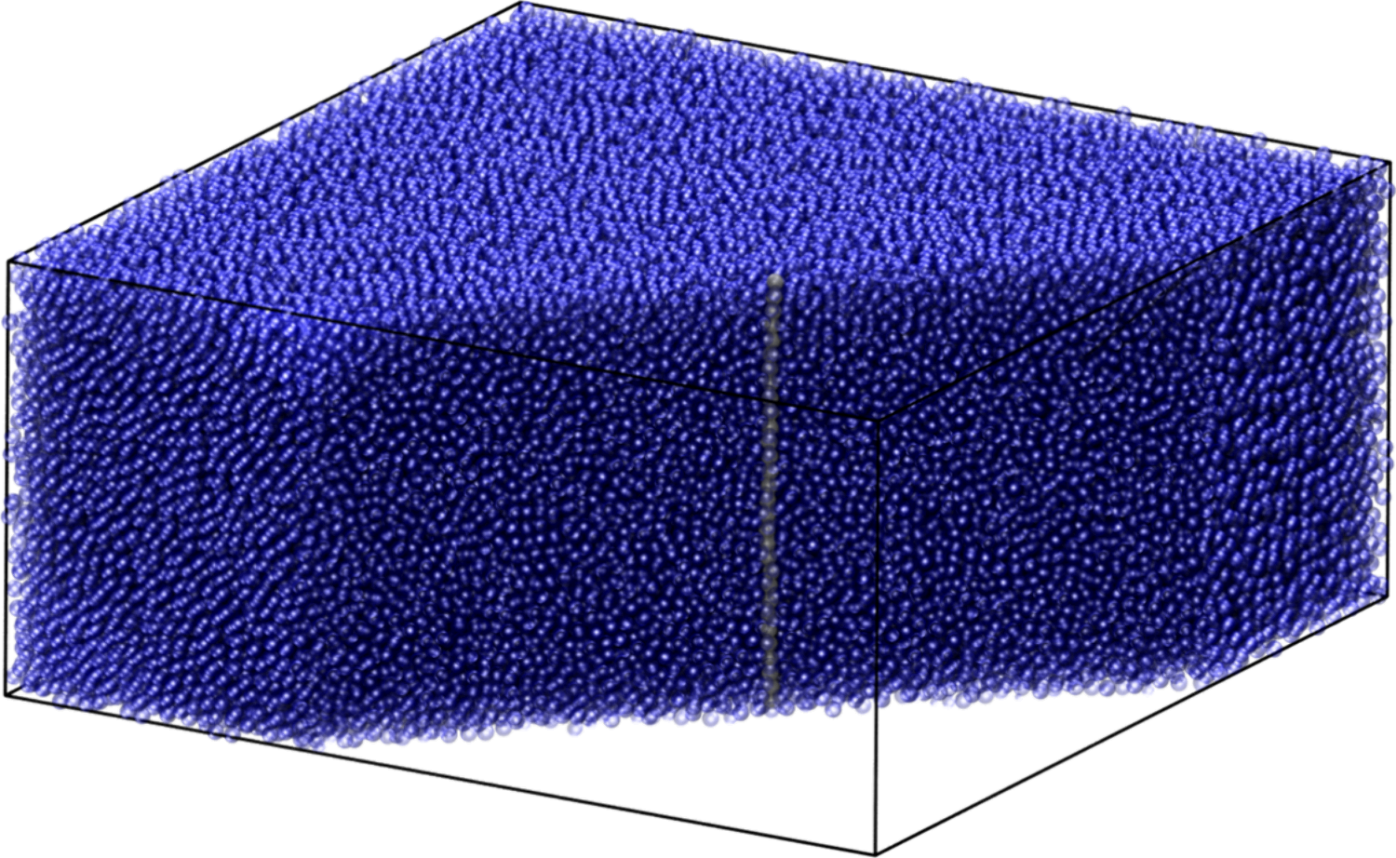
Simulated system setup in dimensions of 67 σ × 67 σ × 30 σ. A solvated 30-mer polymer is attached to itself through the *z*-coordinate. A portion of the solvent beads is removed for visualization purposes so that the polymer is clearly shown.

MD simulations were carried out with the Large-scale Atomic/Molecular Massively Parallel Simulator (LAMMPS)^‡^ ^63^ using periodic boundary conditions. Long-range electrostatics were treated using a particle–particle particle-mesh solver^64^ with a relative error of 0.0001. The configurations for each system type were equilibrated for 50,000 τ with a time step of 0.005 τ, a constant temperature of either T* = 0.7 or 0.79, and pressure of P* = 0.01 with volume fluctuations in the *x* and *y* axes (semi-isotropic NPT). Considering simulation conditions and parameters from Andreev et al.,^61,62^ the system is at a pressure on the order of 8 bar, which is considered low pressure for an incompressible fluid. A value of T* = 0.7 then corresponds to 278 K, and T* = 0.79 corresponds to 314 K. An additional 10,000 τ of simulation time was used to calculate the equilibrium box dimensions in the *x* and *y* directions for subsequent simulations in the canonical ensemble (i.e., constant volume and temperature, NVT), 67 σ × 67 σ × 30 σ for T* = 0.7 and 69 σ × 69 σ × 30 σ for T* = 0.79. After 50,000 τ of equilibration in NVT, a production run of 10,000,000 τ was used for the analysis in our work, as dynamic properties are most reliably represented in the NVT and microcanonical (i.e., constant volume and energy, NVE) ensembles.^65,66^ The Nosé–Hoover^67,68^ thermostat and barostat were utilized with respective dampening factors of 1.0 and 10.0, where applicable. The analysis results of this work were produced in LAMMPS^‡^ ^63^ with the aid of MDAnalysis^‡^ ^69,70^ and the *alchemlyb* ^‡^ ^71^ package.

### Free Energy Calculations

The solvation free energy, Δ*G*_Solv_, is defined as the free energy difference between a solute in solution minus the free energy of the solute in a vacuum and of pure solvent. This work computes Δ*G*_Solv_ using MBAR^57,58^ through the *alchemlyb* ^‡^ ^71^ package, which leverages the *pymbar^‡^* ^72^ package. The basic procedure aims to decouple the interactions between the solute and solvent in a reversible manner such that the free energy may be extracted. We have contributed a module to *alchemlyb* that will read in LAMMPS dumps files, where those files were produced with input scripts generated by the package produced for this work, *generate_alchemical_lammps.* ^73^ MBAR may be used to compute the free energy difference between adjacent states as the interaction potential between the polymer and the solvent scale from fully applied to zero interaction. The free energy of solvation for the polymer was calculated using the soft LJ potential shown in eq 1.

$$U=\lambda^{n}4\epsilon \left\{\frac{1}{{\left[\alpha_{\mathrm{LJ}}(1-\lambda )+{\left(\frac{r}{\sigma }\right)}^{6}\right]}^{2}}-\frac{1}{\alpha_{\mathrm{LJ}}(1-\lambda )+{\left(\frac{r}{\sigma }\right)}^{6}}\right\}$$ 1

Here, λ serves to scale the LJ potential, where a value of λ = 1 produces the full LJ potential and a value of 0 corresponds to a vanishing interaction strength at all distances. The number of windows to scale the potential from 1 to 0 is usually taken to be linearly spaced, so the parameter, *n*, scales their effect to be exponential such that there are fewer windows close to λ = 1 and a higher density of sampling near λ = 0. The parameter α_LJ_ serves to soften the asymptotic repulsive potential as *r* goes to 0. The hyperparameters *n*, α_LJ_, and the number of windows to scale λ from 1 to 0 should be chosen to ensure that this process remains reversible. With this functional form, we found fast convergence when *n* = 3, and α_LJ_ = 0.6, and 25 windows varied λ between 1 and 0. Each window consisted of three phases: a ramp from the previous λ-value to the next over a period of 5,000 τ, an equilibration phase for 5,000 τ, and a production phase for 10,000 τ. These hyperparameters were determined after an assessment of their influence on the solvation free energy of an LJ dimer. The results of this exercise were generated with the aid of iprPy^74^ and may be found in Section S2 of the Supporting Information.

In order to assess the change in solvation free energy with respect to ϵ_PS_, the cross-interaction between polymer and solution (both solvent and salt) was varied using the same methods as Δ*G*_Solv_, but ϵ_PS_ is varied directly in the LJ equation in steps of 0.1 from ϵ_PS_ = 0.4 to 2.0.

### Kirkwood–Buff Integrals

Kirkwood–Buff theory provides a means of calculating the average excess/depletion per unit density of molecule *j*, around molecule *i*, from the pair radial distribution function (RDF) through the computation of Kirkwood–Buff integrals (KBI), *G*_*ij*_^∞^.^75,76^ A combination of values for *G*_*ij*_^∞^ among the components of the relevant system may be used to calculate the partial molar volumes, isothermal compressibility, or chemical potentials.^75–78^ In effect, they provide useful information about the affinity between components.^75^ Equation 2 shows the expression to compute *G*_*ij*_^∞^, but because Kirkwood–Buff theory is defined for the grand canonical ensemble, it is not applicable to MD simulations directly. There are two corrections to account for the difference in ensemble when using MD simulations: finite size effects and the thermodynamic implications of a closed system.^76,79^ Although eq 2 is accurate as the volume becomes infinitely large, it is a simplification of a double integral that is not rigorously valid for a closed system, which results in a deviating behavior in small systems. Krüger and Vlugt derived a rigorously valid single integral by adding a geometry based weighting function to the integrand.^80,81^ Alternatively, eq 3 shows an alternative solution from Lockwood and Rossky^82^ to calculate *G*_*ij*_(*r*) in the canonical ensemble, at some value of *r* (with a maximum limited by the system size), and *V* is the simulation box volume. We found that our simulated system was large enough that the method of Lockwood and Rossky produced similar results to that of Krüger and Vlugt, so this work applies the former. Note, however, that finite size corrections are recommended when applying the latter:^1^

$$G_{ij}^{\infty }=4\pi \int_{0}^{\infty }(g_{ij}(r)-1)r^{2}dr$$ 2

$$G_{ij}(r)=\frac{4\pi \int_{r^{\prime }<r}(1-g_{ij}(r^{\prime })){r^{\prime }}^{2}dr}{1-\frac{1}{V}4\pi \int_{r^{\prime }<r}g_{ij}(r^{\prime }){r^{\prime }}^{2}dr}$$ 3

Note that in eq 3, *G*_*ij*_(*r*) is defined as a function of *r* that should converge to *G*_*ij*_^∞^. One might also define *G*_*ij*_(*r*_cut_) to define the average excess/depletion density between components *i* and *j* within the radius, *r*_cut_.^76^ At some large value of *r*_cut_, *G*_*ij*_(*r*) is expected to converge to *G*_*ij*_^∞^, given that there is a large enough system size so that the solvent beyond *r*_cut_ acts as a particle bath, thus allowing the volume within *r*_cut_ to act like an open system. However, it is known that this expression is difficult to evaluate because of the oscillations present in the RDF over long length scales. Lockwood and Rossky^82^ used the observation that an RDF generated from an alternative choice in the molecular center would yield the same KBI and presented a method of generating a spherical distribution function (SDF) as an average RDF over the alternative molecular centers in a sphere. Their work has since been applied to produce the spherical distribution with the expression:^83–85^

$$\rho \left(\overset{⇀}{r};\xi \right)=\frac{\int_{\left|\overset{⇀}{r}\prime \right|<\xi }\rho \left(\overset{⇀}{r}+{\overset{⇀}{r}}^{\prime }\right)d{\overset{⇀}{r}}^{\prime }}{\frac{4}{3}\pi \xi^{3}}$$ 4

This expression has been used to determine the KBI of functional groups in small solvents, but because our application is for isotropic single bead functional groups, we use the following single integral form derived in Section S4 of the Supporting Information:

$$\rho^{\mathrm{Sph}}(r;\xi )=\frac{\int_{-\xi }^{\xi }\rho (r+r^{\prime })P(r^{\prime },r,\xi )dr^{\prime }}{\frac{4}{3}\pi \xi^{3}}$$ 5

$$P\left(r^{\prime },r,\xi \right)=\pi \frac{r+r^{\prime }}{r}\left(\xi^{2}-{r^{\prime }}^{2}\right)$$ 6

where ξ is the spherical smoothing radius, ρ is the density of the system in that volume element, and *P*(*r* ^′^, *r*, ξ) is a weighting function based on the surface area within the spherical smoothing radius that is at the distance, *r* + *r* ^′^, corresponding to the value of ρ­(*r* + *r* ^′^). The system size studied in this work achieves convergence with a spherical smoothing radius of ξ = 0.8 σ, so that the effective *G*_*ij*_^∞^ is taken to be the average *G*_*ij*_(*r*) between 7 σ and 10 σ for the resulting SDF.

In the interest of quantifying ion effects, we adopt use of the preferential binding parameter, Γ_PI_^∞^, which is used in the assessment of cosolvent effects around biomolecular solutes.^84,86,87^ The expression is given by eq 7 where ρ_I_ is the number density of ions:

$$\Gamma_{\mathrm{PI}}^{\infty }=\rho_{I}(G_{\mathrm{PI}}^{\infty }-G_{\mathrm{PS}}^{\infty })$$ 7

Through this metric, we ascertain a preferential interaction parameter that encompasses bulk solution thermodynamics of the ions (cosolvent) in addition to ion preferential interactions locally with the solute.

With the unique constrained geometry of our simulations, the cylindrical symmetry becomes troublesome with the isotropic assumptions of the original Kirkwood–Buff theory. For that reason, we leverage the cylindrical symmetry to generate a cylindrical distribution function (CDF), eq 8, with reference to the distance between the polymer and either the solvent or ions to produce a KBI for cylindrical geometries (eq 9):

$$g_{\mathrm{CDF},ij}(r)=\frac{\left\langle N_{j}(r)\right\rangle }{2\pi rl\Delta r\rho }$$ 8

$$G_{\mathrm{CDF},ij}^{\infty }=2\pi l\int_{0}^{\infty }(g_{\mathrm{CDF},ij}(r)-1)rdr$$ 9

where *l* is the length of the simulation box in the *z*-coordinate, ρ is the number density of particles in the system and ⟨*N*(*r*)⟩ is the average number of particles in a volume element at some distance from particle type *i*. Because such a geometry has not been entertained in the works applying the method of Krüger and Vlugt, we find further need for the method of analyzing a closed system with adjustments for cylindrical geometry in the method of Lockwood and Rossky, but without the application of spherical smoothing as we presented for RDF-based KBIs. For this case, an average was taken between the peaks in *G*_*CDF,ij*_(*r*) from 3.2 to 4.2 σ.

## Results and Discussion

Changes in the cross-interaction energy between the polymer and the solvent are expected to change the dynamics of the solvent in an extended region around the polymer, i.e., in a DHL. Such a region has only recently been shown to manifest on the ps time scale using the Debye–Waller parameter^9,34^ and the vibrational density of states.^88^ The Debye–Waller parameter, the MSD of the solvent (⟨*u* ^2^⟩) on the caging time scale, 0.8 τ (Figure S4), is a metric that has been utilized in studies of polymeric glass-forming liquids to quantify mobility gradients around inorganic nanoparticles,^31^ and more recently in studies of solvate systems.^9,32,33^ Although trends in ⟨*u* ^2^⟩ have been related to density correlations such as with the primary peak of the RDF,^34^ in a previous atomistic study, the impact of a polymer on solvent density was not nearly as extended as the impact captured by ⟨*u* ^2^⟩.^9^ This observation concurs with previous simulation studies of proteins, where computed metrics indicate that a solute only influences up to two hydration layers from the macromolecular solute,^89–91^ while THz spectroscopy and other methods (see discussion in the introduction of ref 9) probing the water dynamics show a more extended effect. Figure 2a shows the CDF (cylindrical analogue to the RDF) for the solvent around the straight polymer chain. There is a direct relationship between the polymer–solvent interaction energy, ϵ_PS_, and peak heights (and minimum depths). As ϵ_PS_ increases, the more pronounced features indicate a more ordered system, with an increasingly strong attraction between molecular species. Turning to ⟨*u* ^2^⟩ (Figure 2b), we observe a richer behavior where a low value of ϵ_PS_ = 0.4 results in increased dynamics of the solvent. This increase in short time dynamics around our hydrophobic polymer exceeds the increase in mobility of solvent around the chaotropic ion, Cs^+^, itself, as demonstrated in a previous work.^34^ With a strong interaction of ϵ_PS_ = 2.0 in our hydrophilic polymer, the mobility of the solvent decreases, such as in the case of solvent around kosmotropic Na^+34^ or charged polybetaine simulations.^9^ Of the limited number of studies that have applied this type of analysis to solvated systems, the DHL gradients (shown unscaled in the Section S6 of the Supporting Information) resemble the ⟨*u* ^2^⟩ values around single ions in infinite dilution (Figure 4a in ref 34) with well-defined peaks and minima, indicative of an isotropic system such as designed in this work. To more clearly visualize the extent of mobility increases and decreases with a change in ϵ_PS_, the inlay of Figure 2b shows the same gradient scales by the ϵ_PS_ = 1 case. These results show that the simulation criteria that we have chosen can capture DHLs of both hindered and accelerated types.

**Figure 2:**
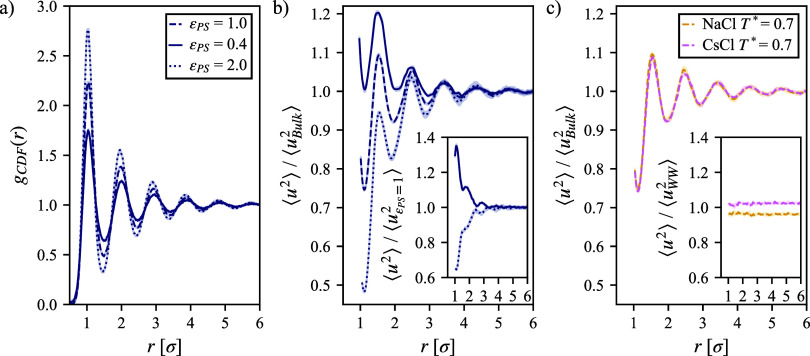
(a) Cylindrical distribution function (CDF) of solvent around an infinitely long straight-chain polymer at T* = 0.7 without the presence of salt for variable cross-interaction parameters. (b) Debye–Waller parameter ⟨*u* ^2^⟩ at T* = 0.7 without salt and scaled by bulk value with an inlaid plot of these same profiles scaled by the gradient for ϵ_PS_ = 1. (c) Relative Debye–Waller parameter ⟨*u* ^2^⟩ for 1 mol/L NaCl and CsCl solution scaled by their respective bulk values. Inlay represents ⟨*u* ^2^⟩ profiles scaled by the no salt solution case. Full-temperature and salt-dependent trends are found in Section S6 of the Supporting Information. Shaded regions represent the standard deviation over the three independent boxes and may be smaller than the plotted trend.

Notice that the influence of 1 mol/L CsCl and NaCl on ⟨*u* ^2^⟩ (Figure 2c) aligns with expected chaotropic and kosmotropic trends previously reported around alkali salts; however, there is apparently little change in the radial dependence of ⟨*u* ^2^⟩ around the polymer relative to the bulk material. As we have seen before,^34^ kosmotropic ions such as Na^+^ decrease the mobility of the solvent and chaotropic ions such as Cs^+^ give rise to the opposite effect. We see that that while ϵ_PS_ greatly influences the mobility gradient around the polymer, the addition of either kosmotropic or chaotropic salts has a uniform effect on ⟨*u* ^2^⟩ relative to the surrounding bulks solvent, as shown in the inlay of Figure 2c. We thus see no additional specific ion effects on the DHL in this case. This finding surprised us given the observed change in the distribution function of ions around the polymer indicated in Figure 3a. The primary peak height represents relative strength of interactions, and despite there being no energetic preference between the polymer with the cation versus the solvent, the peak heights are significantly different. Figure 3b illustrates this point where the CDF for the solvent without salt is subtracted from the CDF of the ions given a set of system conditions. It is apparent from Figure 3b that there is an excess of CsCl and depletion of NaCl close to the polymer and that the effect increases when the polymer is more hydrophobic. This result is intriguing considering that the interaction energy is equal and cannot explain the excess and depletion of the ions: some other mechanism is at play.

**Figure 3:**
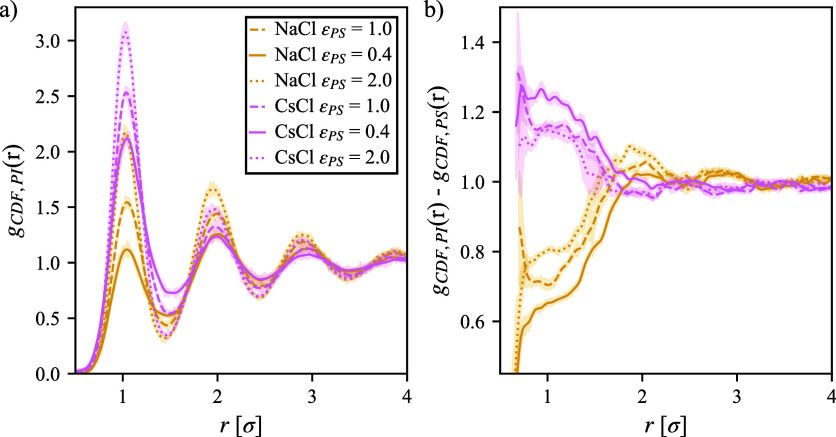
(a) Cylindrical distribution function (CDF) of Na^+^ and Cs^+^ around a long rod-like polymer. (b) Difference in CDF between solvent and ions around the polymer; note that a Gaussian filter with a standard deviation of five was applied to improve readability. Uncertainty intervals represent the standard deviation over three independent simulation boxes, where large uncertainties indicate a region with poor sampling of solvent molecules or may be smaller than the plotted trend.

Our initial hypothesis was that the DHL around a polymer should have an influence on the free energy of solvation of the polymer. Such an extended region of solvent has been observed experimentally for proteins^16,21,22^ and has also been shown in atomistic simulations of synthetic polar polymers such as polybetaines.^9^ Although macromolecular proteins and brushy polybetaines are expected to have a larger influence due to surface area, this study sets the groundwork for isolating the effects of hydration and salt interactions without the convolution of configurational changes. We will then begin by illustrating this point in Figure 4a, where an increase in the potential-well depth for the polymer–solvent interaction potential, ϵ_PS_, signifies an increased affinity between the polymer and surrounding solvent and so is accompanied by an expected decrease in the solvation free energy, Δ*G*_solv_. The solvation free energy exhibits significant temperature-dependent changes, illustrating an entropic change in the system, as supported by the separation of the entropic contribution in Figure 4c. However, on the basis of standard Flory–Huggins theory,^37,38^ there should be no entropic change to the free energy given that we have constrained the polymer chain dimensions (see Section S1 of the Supporting Information). Figure 4b illustrates a dependence of the enthalpy on ϵ_PS_, although less than the entropic changes (Figure 4c). The changes in enthalpy with respect to temperature reflect the scaling by T*, leaving the temperature dependence restricted to the entropic term. These results indicate large deviations from standard Flory–Huggins theory, which neglects the effect of hydration.^37,38^

**Figure 4:**
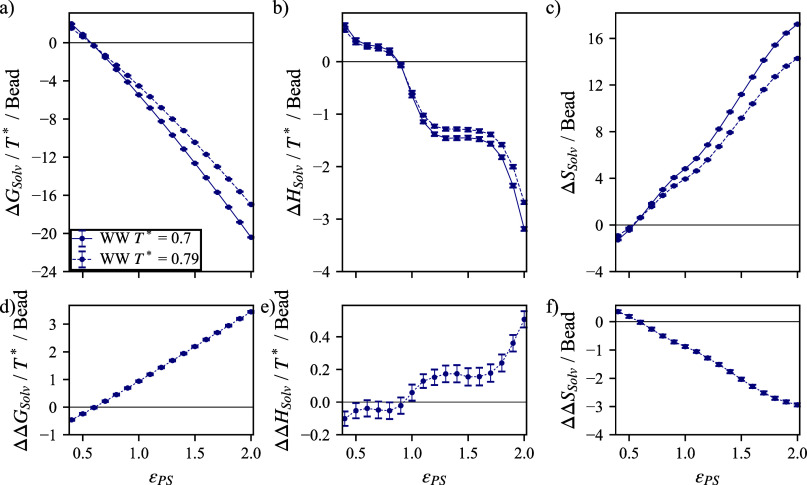
For a system without salt, the (a) free energy, (b) enthalpy, and (c) entropy of solvation, i.e., free energy to decouple the polymer and solvent, are plotted with respect to the interaction energy between polymer and solvent for two temperatures. The difference in solvation (d) free energy, (e) enthalpy, and (f) entropy between low and high temperatures illustrate temperature-dependent solvation behavior. Uncertainty intervals may be smaller than data markers, which represent the standard deviation over three independent simulation boxes.

There is a shift in the crossover from positive to negative values of Δ*G*_solv_, toward higher values of ϵ_PS_ that suggest a change in either the effective polymer–solvent interactions or effective solvent–solvent interactions. As derived in Section S1 of the Supporting Information, the enthalpy of mixing due to the opportunity of cost of hydrating the polymer equals

$$U_{\mathrm{mix}}=(z-z_{\mathrm{pp}})\phi_{P}\left[\varepsilon_{\mathrm{PS}}^{\mathrm{FH}}-\frac{\varepsilon_{\mathrm{SS}}^{\mathrm{FH}}}{2}\right]$$ 10

where *z* is the total coordination number of the polymer, *z*_pp_ is the coordination number of the polymer with itself, ϕ_P_ is the volume fraction of the polymer, and ε_PS_^FH^ is the Flory–Huggins lattice model interaction energy between the polymer and solvent; likewise, ε_SS_^FH^ represents the interactions between the solution beads. It is known that the Flory–Huggins interaction parameter is proportional to the negative of an LJ potential-well depth parameter, i.e., ε^FH^ ∝ – ϵ. However, developing such a simple expression for ε_SS_^FH^ is not as straightforward for salt solutions, as solvent–solvent, solvent–ion, and ion–ion interactions must all be accounted for, which would involve including Coulombic contributions. Recall from Table 1 that all values of ϵ are equal to 1, except ϵ_PS_ and ϵ_CS_, and that for our model, ϵ_PS_ = ϵ_PC_ = ϵ_PA_. This model avoids preferential interaction between the polymer and ions, which would convolute our analysis of the DHL. Thus, while ε_PS_^FH^ may be considered a direct analog of ϵ_PS_, ε_SS_^FH^ must account for the solvent–solvent, solvent–ion, and ion–ion nonbonded interactions and ion–ion charged interactions.

A direct analog of ε_SS_^FH^ would be an effective value of ϵ_SS_^eff^ accounting for ion–ion charges and nonbonded interactions with ϵ_SS_ = ϵ_AS_ = ϵ_AC_ = ϵ_AA_ = ϵ_CC_ = 1, while ϵ_CS_ is 1.25 and 0.85 for Na^+^ and Cs^+^, respectively.^61^ Kosmotropic NaCl increases the viscosity and decreases the diffusivity of water, while chaotropic CsCl has the opposite effect. This same trend observed in Figure 2c for ⟨*u* ^2^⟩ aligns with our previous work suggesting these effects are linked to changes in the cohesive energy density (CED) of the solution.^34^ CED is the intermolecular energy of the system.^92,93^ In a pure solvent solution, without polymer, there is no intramolecular energy and the CED from simulations is simply the potential energy times the number density. We previously showed that the CED aligns with kosmotropic/chaotropic trends.^34^ These trends do not have a significant affect Δ*G*_solv_ of the polymer in our system, where the chain is constrained and the interaction energy between the polymer and ions is equal to ϵ_PS_. These two simplifications exclude general trends from direct experimental analogy but deconvolute contributions from polymer–polymer interactions and explicit preferential interactions between solution components (solvent, cation, or anion) to identify the source of known specific ion effects. The Debye–Waller parameter scales with chaotropic and kosmotropic trends but does not greatly affect the ⟨*u* ^2^⟩ profile shape. Similarly, the impact on Δ*G*_solv_/T* per bead is slightly increased by 4.3 × 10^–4^ in a 1 mol/L NaCl solution and decreased by −3 × 10^–4^ in a 1 mol/L CsCl solution at T* = 0.7 (Figure S2), with a similar trend and magnitude at the higher temperature. Specific ion effects on the DHL are subtle in their impact on Δ*G*_solv_/T*, so known specific ion effects are expected to manifest with relaxation of configurational constraints and/or assignment of ϵ_PC_ ≠ ϵ_PS_. This is despite the increased CsCl density near the polymer and NaCl depletion. Sources of variation in ϵ_SS_^eff^, other than ion–solvent interactions, would stem from effective changes in polymer–solution interactions due to the DHL character, either hindering or enhancing mobility as quantified with ⟨*u* ^2^⟩.

In order to quantify the effective intermolecular affinity, ϵ_PS_^eff^ and ϵ_SS_^eff^, we turn to Kirkwood–Buff integrals, *G*_*ij*_^∞^, which relate to thermodynamic properties of a solution, including the chemical potential^75–78^ and second virial coefficients.^94^ This quantity is calculated via simulation through the RDF, a structural measure that captures the response to an effective affinity with a positive value representing density excess or a negative value representing depletion. Because our polymer is constrained to a straight geometry, the values presented here are higher than those of a freely moving polymer in dilute solution. However, without the excluded volume effects on the RDF caused by polymer–polymer interactions, we may clearly compare *G*_*ij*_^∞^ values between systems with varying ϵ_PS_ to study the effect of the DHL without the compounding effect of a collapsed polymer limiting solvent access in the hydrophobic case. With this metric in hand, we will review the implications of changes in ϵ_PS_^eff^ and ϵ_SS_^eff^.

Overall, there is apparently a significant change in ϵ_PS_^eff^ aligning with ϵ_PS_ as expected but with quantitative specific ion, cosolvent preferential binding parameters. Notice in Figure 5a,b that *G*_CDF,PI_^∞^ and *G*_CDF,PS_^∞^ are negative, indicating a density depletion close to the polymer, when ϵ_PS_ = 0.4 or 1.0, and only reach a neutral effective affinity with the solution when ϵ_PS_ = 2.0. It is possible that this observation arises from our constrained, stiff chain geometry, where the polymer cannot bend to allow for efficient packing. We also observe specific ion effects with a difference in *G*_CDF,PI_^∞^ versus *G*_CDF,PS_^∞^. Since the ions can be viewed as a cosolvent, we apply the preferential binding parameter,^84,86,87^ Γ_I_, to produce a metric to capture the depletion of NaCl and the excess of CsCl relative to the solvent, as observed in Figure 3b. It is apparent that as the solution interaction becomes stronger, with high hydrophilic ϵ_PS_ = 2.0 (recall that Li^+^ interactions with solvent are 1.85 for this model^62^), the solvent partitioning becomes negligible, but that at more comparable and low values of ϵ_PS_, this partitioning occurs, despite the same interaction energy existing between the polymer and solvent. There is a known affinity of Cs^+^ to partition near surfaces.^95^ It is apparent from our constrained system that this behavior is only explained by the presence of the DHL. Such attraction seems reminiscent of the *law of matching water affinities*, which suggests that monatomic ions in the chaotropic/kosmotropic series interact in contact, solvent-separated, or two solvent-separated ion pairs depending on the similarity of the strengths in their hydration.^95,96^ Indeed, if appropriate polymer–ion interaction parameters were assigned, then we expect that specific ion effects would result in more prevalent changes in Δ*G*_solv_, thus providing an indirect influence of the DHL on specific ion effects.

**Figure 5:**
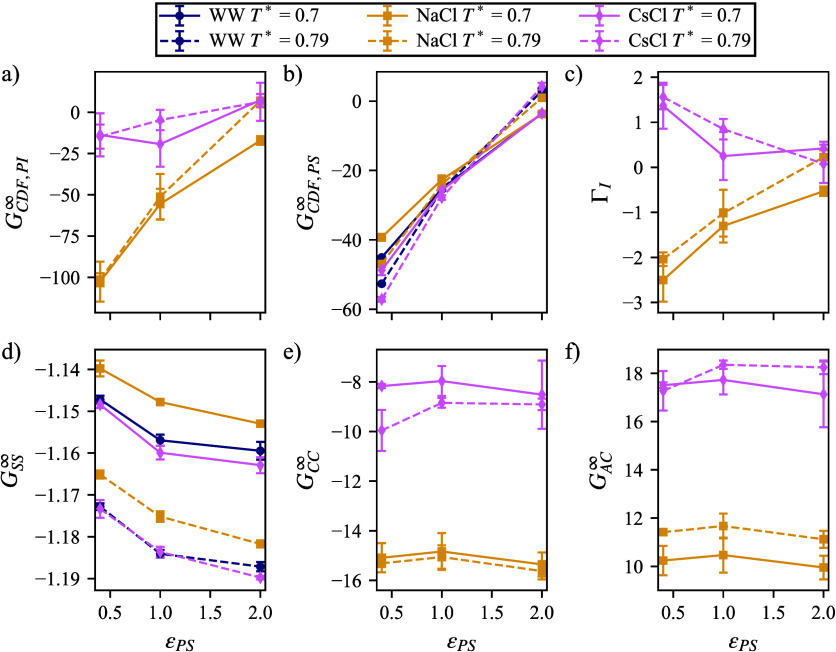
Kirkwood–Buff integrals using the CDF between (a) the polymer and ions (both anion and cation), (b) the polymer and solvent, and (c) the interaction parameter of the ions relative to the solvent. These can be compared to Kirkwood–Buff integrals using the SDF between (d) the solvent and solvent, (f) the cation and cation, and (g) the anion and cation. Uncertainty intervals represent the standard deviation over three independent simulation boxes and may be smaller than the data markers.

As one might expect, given the system size with a polymer volume fraction of ϕ_P_ = 3 × 10^–4^, there are not significant changes in *G*_SS_^∞^, *G*_CC_^∞^, or *G*_AC_^∞^ shown in Figure 5d, Figure 5e, and Figure 5f respectively, representing the effective affinity between solvent–solvent, cation–cation, and anion–cation; in the bulk, however, close to the polymer, we find significant changes in ϵ_SS_^eff^. Although changes in *G*_SS_^∞^ are statistically dependent on ϵ_PS_, which is impressive given the volume fraction, there is a richer change when we focus on the DHL. Because Kirkwood–Buff integrals are defined from zero to an infinitely large distance, we will compare *G*_SS_(*r*) values at some cutoff distance (*r*_cut_ = 1.68 σ). Representation of the DHL is achieved through restricting the RDF calculation between solution beads in the DHL (within 2.5 σ for largely impacted solution beads within the DHL of 3 σ) and all solution; then, the SDF is applied. Figure 6 illustrates large changes between *G*_*ij*_(*r*) for the bulk and the DHL. An example of the change in *G*_AC_(*r*) with respect to distance from the polymer is shown in the inlay of Figure 6c. It becomes apparent that while *G*_SS_^rcut^ is constant with respect to ϵ_PS_ in the bulk (Figure 6a), in the DHL, *G*_SS,DHL_^rcut^ (Figure 6d) the trends align qualitatively with changes in *G*_PS_^∞^ shown in Figure 5b. Effective affinities between cations appear to be largely unchanged; conversely, there is a dramatic difference between the effective affinities between anion and cation. *G*_AC_^rcut^ versus *G*_AC,DHL_^rcut^ are shown in the comparison of Figure 6c,f. While neither case has a dramatic dependence on ϵ_PS_, the expected positive affinity between Cl^–^ and either Na^+^ or Cs^+^ becomes significantly unfavorable in the DHL. The divergence in *G*_AC_(*r*) is shown in the inlay of Figure 6c. While this dramatic change in effective anion–cation affinity does not manifest in changes in Δ*G*_solv_, it should be kept in mind for future studying with a relaxed constraint on the polymer configuration. It is apparent that within the DHL, ϵ_SS_^eff^ is altered, especially in the case of solvent–solvent interactions following trends in *ϵ*_PS_.

**Figure 6:**
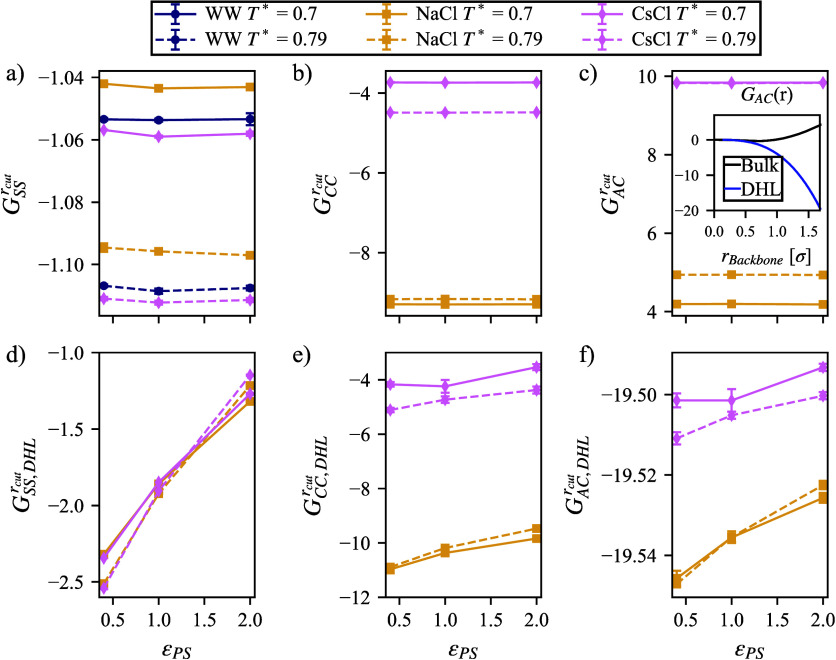
Truncated Kirkwood–Buff integrals at a cutoff. The top row represents values of *G*_*ij*_ at *r*_cut_ = 1.68 σ in bulk solution for (a) solvent and solvent, (b) cation and cation, and (c) anion and cation. The inlay of (c) illustrates how the KBI varies between these two solution environments, resulting in the values at the cutoff reported here. The bottom row represents values taken from solution beads in the dynamic hydration layer (DHL) for (d) solvent and solvent, (e) cation and cation, (f) and anion and cation. Uncertainty intervals represent the standard deviation over three independent simulation boxes and may be smaller than data markers.

Another aspect of applying the conceptual contributions of eq 7 would be to take the effect of the coordination number, *z*, into account. While standard lattice models assume a value of *z* = 6, our simulation results show that there is more variability. While the coordination number between solution beads is fairly consistent in Figure 7b, the coordination between the polymer and solvent is variable with respect to ϵ_PS_. This observation is intuitive, in that a stronger interaction would lead to denser packing around the polymer (and lower mobility). However, whether it be from our constrained, straight-chain geometry, or more generally from the universality of two polymer bead neighbors, the coordination around the polymer is less than the coordination for the solution. If the coordination of solvent around the polymer is defined to be *z*_PS_ plus *z*_PP_ = 2 (for a coordination cutoff of 1.5 σ with a chain of 1 σ beads), then it is only at the highest value of ϵ_PS_ that the coordination of the polymer reaches the level of coordination around the solvent (*z*_SS_ ≈ *z*_PS_ + 2).

**Figure 7:**
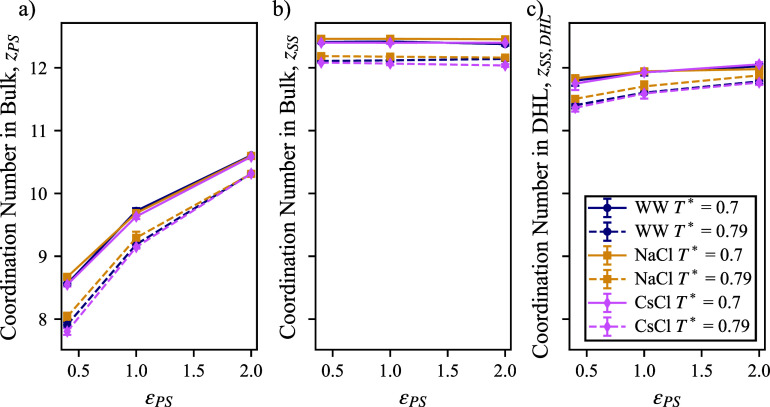
Coordination number (a) around for polymer and solution (including solvent and ions) and (b) between solution and solution. These coordination numbers were taken at the first minimum of the RDF, which was at (1.57 ± 0.01) σ and (1.51 ± 0.01) σ for the two cases, respectively. (c) Coordination number between solution and solution beads in the dynamic hydration layer within 2.5 σ of the polymer. Uncertainty intervals represent the standard deviation over three independent simulation boxes and may be smaller than data markers.

However, in isolating the solvent within 2.5 σ around the polymer (within the DHL of 3 σ), we see the coordination dependence on ϵ_PS_. The coordination number between solvent and solvent is reduced most dramatically when ϵ_PS_ = 0.4, where the dynamics are accelerated, and remains closest to the bulk value for the high value of ϵ_PS_ = 2.0. It is interesting that even the energetically neutral case (ϵ_PS_ = 1.0) exhibits a reduction in coordination, thus adding a penalty for a solvent bead to enter the DHL. Although we observe the expected decrease in enthalpy with increasing ϵ_PS_, an apparent step function is not expected. Unfortunately, our trends in coordination number were not recorded at the same granularity with which we obtained our free energy trends, but we could speculate that the step size changes in enthalpy (Figure 4b) could relate to changes in coordination. We expect that the lower coordination around the polymer for the hydrophobic (ϵ_PS_ = 0.4) versus the hydrophilic (ϵ_PS_ = 2.0) polymer may cause the changes in enthalpy in Figure 4b.

## Conclusions

This work seeks to characterize the free energy effects of introducing a polymer into solution, demonstrating the formation of a DHL and the changes in entropy depending on the strength of the interaction between the polymer and solvent, ϵ_PS_. This work involves a system where all potential-well depths are set to unity except appropriate ion–solvent and polymer-soluiton interactions. ϵ_PS_ varied between a hydrophobic case (i.e., ϵ_PS_ = 0.4), an energetically neutral case (i.e., ϵ_PS_ = 1.0), and a hydrophilic case on the order of a charged polymer (i.e., ϵ_PS_ = 2.0). The effects of these interaction parameters on solvation are isolated through the conformational restriction of the polymer as an infinitely long chain connected to itself by periodic boundary conditions. It was shown that with a hydrophobic interaction energy (i.e., ϵ_PS_ = 0.4), the entropy of mixing was positive, the DHL exhibited accelerated dynamics, and that there were fewer solvent–solvent and polymer–solvent interactions within the DHL. When a hydrophilic interaction energy was used (i.e., ϵ_PS_ = 2.0), the entropy of mixing was negative (indicative of LCST behavior), the DHL showed hindered mobility, and the coordination between solvent–solvent and polymer–solvent in this layer was similar to the bulk. This is despite the restriction to a simulated system where the entropic contributions considered by Flory–Huggins theory are zero (as derived in Section S2 of the Supporting Information). This work shows interesting trends in the enthalpy of solvation, although its contribution is far outweighed by the entropy of solvation. Given the constrained geometry of our polymer, this dramatic change in the entropy of solvation must stem from the presence of the DHL. While Flory–Krigbaum theory^39^ suggests such a term might vary linearly with respect to temperature and polymer concentration, our results show additionally a largely linear dependence with respect to the polymer–solution interaction, demonstrating little change with respect to solution–solution interactions as represented by the addition of salts. The development of an analytical expression to represent the entropic contribution of the DHL and changes in coordination would serve to improve the analytical modeling of dilute solutions.

Although this work did not demonstrate specific ion effects in the free energy, enthalpy, or entropy of solvation, other properties demonstrate specific ion changes that would influence the thermodynamics of the system after the removal of two simplifications in this work. This system is constrained to an infinitely long chain to remove the convoluting effect of polymer–polymer interactions and provides an isotropic system from which to study polymer hydration. Allowing for a freely moving chain would then allow for excluded volume effects on the hydration, allowing the system to minimize or maximize the chain dimensions in accordance with the thermodynamic impact of the DHL. The other simplification of setting ion–polymer interactions equal to solvent–polymer interactions likely serves a large role in the lack of specific ion effects observed in the thermodynamic contributions. Indeed, our analysis of the CDF and Kirkwood–Buff integrals suggests there is higher ion affinity of Cs^+^ and lower for Na^+^ despite the equal interaction energy between them or the solvent and the polymer, aligning with known phenomena of partitioning of Cs^+^ close to surfaces.^95^ If the nature of a hydrophobic hydration layer provided a means of indirect attraction, as our results show, then this seems akin to the observations leading to the *law of matching water affinities* ^95,96^ of monatomic and polymer ions, suggesting that the closeness of interaction is dictated by similar hydration layer characteristics (i.e., mobility). Nonetheless, adding specific ion-polymer interactions is then expected to influence thermodynamic properties with this variation in number density for the solution components. Additionally, values of Kirkwood–Buff integrals in the DHL suggest that the affinity between cations and anions becomes unfavorable close to the polymer. Thus, salt partitioning dynamics is expected to play a role in polymer chain dimensions and dynamics.

## Supplementary Material



## Notes

Rederivation of Flory–Huggins theory and an adaptation for the constrained infinitely long-chain system presented in this work; influence of alchemical hyperparameters on the resulting free energy of solvation; comparison of the free energy, enthalpy, and entropy of solvation for pure solvent, 1 mol/L NaCl, and 1 mol/L CsCl solutions; derivation of the spherical distribution smoothing function applied in this work; determination of the caging relaxation time used to define ⟨*u* ^2^⟩; and polymer–solution cylindrical distribution function and solution Debye–Waller parameter profiles around the polymer (PDF)
