# Supplementary material for: Hydration Contribution to the Solvation Free Energy of Water-Soluble Polymers
Source: J Phys Chem B. 2025 Jun 3;129(26):6548–60. doi: 10.1021/acs.jpcb.5c01009 (PMC12235621; doi:10.1021/acs.jpcb.5c01009)
Supplement: Supplementary file 1 [file jp5c01009_si_001.pdf]

Supplemental Information:
Hydration Contribution to the Solvation Free Energy of Water-Soluble Polymers

Jennifer A. Clark[1,] and Jack F. Douglas[1]

[1]Materials Science and Engineering Division, Material Meas1urement Laboratory,

National Institute of Standards and Technology, Gaithersburg, Maryland 20899

S1. Lattice Theory for Solvated Infinitely Long Rod-like Polymers

We first review the derivation of the standard Flory-Huggins (FH) expression for the free energy of polymer solutions in a solvent, and adapt this idealized model approach for an solvated infinitely long polymer. We highlight predictions of this mean filed model of polymer solutions and in the left panel we specialize the derivation corresponding to the system that we study computationally. Note that because of the assumed geometry of our polymer system, we completely neglect polymer-polymer interactions.

Symbols in the tables below:

ϕ_i: Volume fraction of component

N_i: Number of segments in molecule

n_i: Number of molecules in the system

z: total coordination number

z_p: polymer-polymer coordination number

m_{ij}: is the number of contacts between two types of sites

M: total number of sites (i.e., volume)

k_B: Boltzmann constant

T: Temperature

1.1 Summary of Standard Flory-Huggins Theory Results and Specialization to Our Model

1.1.1 Flory-Huggins (FH) Free Energy

The free energy of mixing for a lattice model is the sum of the entropy and the heat of mixing:[1]

$$\frac{A_{mix}}{Nk_BT} = \frac{U_{mix}}{Nk_BT} - \frac{S_{mix}}{Nk_B}$$

where A_{mix} is the Helmholtz free energy, U_{mix} is the potential energy, and S is the entropy of the system.

This expression involves the following relationships:

Original FH Theory[1–3]	Isolated Infinite Chain
$$\frac{A_{mix}}{Nk_BT} = \chi\phi_P\phi_S + \frac{\phi_P \ln \phi_P}{N_P} + \phi_S \ln \phi_S$$ $$\chi = \frac{z}{k_BT}\left(\epsilon_{PS} - \frac{\epsilon_{PP} + \epsilon_{SS}}{2}\right)$$	$$\frac{A_{mix}}{Nk_BT} = \chi\phi_P + 0$$ $$\chi = \frac{(z - z_p)}{k_BT}\left(\epsilon_{PS} - \frac{\epsilon_{SS}}{2}\right)$$ The configurational entropy is zero for this system with an infinitely straight polymer.

1.1.2 Enthalpic Contribution

<table>
<tr><td>Original FH Theory</td><td>Isolated Infinite Chain</td></tr>
<tr><td colspan="2">Note that $U_i = \sum m_{ij}\epsilon_{ij}$ where m_{ij} is the number of contacts. For a pure solvent, the number of contacts is simply the number of solvent beads times the coordination number, then divided by two to remove duplicate counting.

$$\frac{U_{solvent}}{N} = \frac{z\phi_S}{2}\epsilon_{SS}$$</td></tr>
<tr><td>Similarly, $U_{polymer}$ equals,

$$\frac{U_{polymer}}{N} = \frac{z\phi_P}{2}\epsilon_{PP}$$</td><td>Because the polymer is constrained to be a straight chain, $U_{polymer}$ reduces to the form:

$$\frac{U_{polymer}}{N} = \frac{z_p\phi_P}{2}\epsilon_{PP}$$</td></tr>
<tr><td colspan="2">The enthalpy in solution must then account for the interactions between solvent and polymer.</td></tr>
<tr><td>$$\frac{U_{solution}}{N} = \frac{z\phi_S - m_{PS}}{2}\epsilon_{SS} + \frac{z\phi_P - m_{PS}}{2}\epsilon_{PP} + m_{PS}\epsilon_{PS}$$</td><td>$$\frac{U_{solution}}{N} = \frac{z\phi_S - m_{PS}}{2}\epsilon_{SS} + \frac{z_p\phi_P}{2}\epsilon_{PP} + m_{PS}\epsilon_{PS}$$</td></tr>
</table>

Assume the probability of two beads interacting is completely random, so that $m_{PS} = z\phi_P\phi_s$.We then have,<br>$\frac{U_{solution}}{N} = \frac{z\phi_S}{2}\epsilon_{SS} + \frac{z\phi_P}{2}\epsilon_{PP} + z\phi_P\phi_s\left(\epsilon_{PS} - \frac{\epsilon_{PP} + \epsilon_{SS}}{2}\right)$	Since the number of interactions between polymer beads will not change, the number of polymer-solvent contacts is simply, $m_{PS} = (z - z_p)\phi_P$, we then have,<br>$\frac{U_{solution}}{N} = \frac{z\phi_S}{2}\epsilon_{SS} + \frac{z_p\phi_P}{2}\epsilon_{PP} + (z - z_p)\phi_P\left(\epsilon_{PS} - \frac{\epsilon_{SS}}{2}\right)$
The overall enthalpic term then equals,<br>$\frac{U_{mix}}{N} = \frac{U_{solution}}{N} - \frac{U_{polymer}}{N} - \frac{U_{solvent}}{N} = z\phi_P\phi_s\left(\epsilon_{PS} - \frac{\epsilon_{PP} + \epsilon_{SS}}{2}\right)$<br>$\frac{U_{mix}}{Nk_BT} = \chi\phi_P\phi_s$<br>$\chi = \frac{z}{k_BT}\left(\epsilon_{PS} - \frac{\epsilon_{PP} + \epsilon_{SS}}{2}\right)$	The overall enthalpic term then equals,<br>$\frac{U_{mix}}{N} = \frac{U_{solution}}{N} - \frac{U_{polymer}}{N} - \frac{U_{solvent}}{N} = (z - z_p)\phi_P\left[\epsilon_{PS} - \frac{\epsilon_{SS}}{2}\right]$<br>$\frac{U_{mix}}{Nk_BT} = \chi\phi_P$<br>$\chi = \frac{(z - z_p)}{k_BT}\left(\epsilon_{PS} - \frac{\epsilon_{SS}}{2}\right)$

1.1.3 Entropic Contribution

**Original FH Theory**	**Special Case of Isolated Infinite Rod-like Chains**
Assume a uniform distribution of polymer segments over the entire volume. This expression is derived[3] with the knowledge that $S_{solvent} = 0$, and so the entropic contribution is derived from the solution term:<br>$\frac{S_{solution}}{Nk_B} = \left(\phi_S + \frac{\phi_P}{N_P}\right)\ln(\phi_S + \phi_P) - \phi_S\ln(\phi_S) - \frac{\phi_P}{N_P}\ln\left(\frac{\phi_P}{N_P}\right) + \frac{\phi_P}{N_P}\ln\left(\frac{z}{\sigma}\right) + (N_P - 2)\frac{\phi_P}{N_P}\ln(z - 1) - (N_P - 1)\frac{\phi_P}{N_P}$<br>where σ removes double counting the forward and backward counting of chain ends and so equals 2, however, it is often safely neglected for sufficiently long chains and set equal to 1.	Because our system involves infinitely long chains, the entropy of the polymer is constrained to a 2D plane, where the placement of the polymer beads are constrained by the placement of the first bead of each chain. Because the length of the chain defines the height of the box, the number of placements is equal to the total number of sites in the box, scaled by the length of the chains, M/N_P. The number of sites available for the chain would then be, $x = \frac{N_P n_P + n_S}{N_P}$:<br>$\frac{S_{solution}}{Nk_B} = \ln\left(\frac{N_P n_P + n_S}{N_P}\right)$<br>$\frac{S_{solution}}{Nk_B} = -\ln(\phi_P)$

The entropy for a pure polymer system is taken from $\frac{S_{solution}}{k_B}$ in the case that $\phi_S = 0$:	
$$\frac{S_{polymer}}{Nk_B} = \frac{\phi_P}{N_P}\ln\left(\frac{zN_P}{\sigma}\right) + (N_P - 2)\frac{\phi_P}{N_P}\ln(z-1) - (N_P - 1)\frac{\phi_P}{N_P}$$	$$\frac{S_{polymer}}{Nk_B} = -\ln(\phi_P)$$
The overall entropic term is then: $$-\frac{S_{mix}}{Nk_B} = \frac{S_{polymer}}{k_B} + 0 - \frac{S_{solution}}{k_B} = \frac{\phi_P \ln \phi_P}{N_P} + \phi_S \ln \phi_S$$	$$-\frac{S_{mix}}{Nk_B} = \frac{S_{polymer}}{k_B} + 0 - \frac{S_{solution}}{k_B} = -\ln(\phi_P) + -\ln(\phi_P) = 0$$

1.1.4 Total Free Energy of Mixing Expression

The free energy of mixing for a lattice model is the sum of the entropy and the heat of mixing:[1]

$$\frac{A_{mix}}{Nk_BT} = \frac{U_{mix}}{Nk_BT} - \frac{S_{mix}}{Nk_B}$$

Where A_{mix} is the Helmholtz free energy, U_{mix} is the potential energy, and S is the entropy of the system.

This expression involves the following relationships:

Original FH Theory[4]	Isolated Infinite Chain
$$\frac{A_{mix}}{Nk_BT} = \kappa\left(\mathrm{x}'\frac{1}{V_S}\right)\phi_s + \phi_S \ln \phi_S - \left(\psi - \frac{1}{2}\right)\phi_S\phi_P$$ $$\kappa = \frac{(z-2)}{k_BT}\frac{V_S}{V_P}\left(\epsilon_{PS} - \frac{\epsilon_{PP} + \epsilon_{SS}}{2}\right)$$ where x' is the number of polymer beads, expressions defined in Section S1.2.2 in the volume element δv. All volume elements must be integrated over to generate the free energy landscape.	$$\frac{A_{mix}}{Nk_BT} = \kappa\phi_P - \left(\psi - \frac{1}{2}\right)\phi_P$$ $$\kappa = \frac{(z - z_p)}{k_BT}\left(\epsilon_{PS} - \frac{\epsilon_{SS}}{2}\right)$$ $$\frac{A_{mix}}{Nk_BT} == \left(\kappa - \psi + \frac{1}{2}\right)\phi_P = \chi\phi_P$$ $$\chi = \kappa - \psi + \frac{1}{2}$$

1.1.5 Enthalpic Contribution

Original FH Theory	Isolated Infinite Chain
We first follow the original derivation of Flory-Huggins theory as shown in Section S1.1.2, with an alteration that the volume of the polymer chain and of the solvent is incorporated under a new variable, κ, and the enthalpy change is assessed for each volume element, δv, individually.	

$\frac{U_{mix}}{Nk_BT} = \kappa\left(\mathrm{x}'\frac{1}{V_S}\right)\phi_s$<br>$\kappa = \frac{(z-2)}{k_BT}\frac{V_S}{V_P}\left(\epsilon_{PS} - \frac{\epsilon_{PP}+\epsilon_{SS}}{2}\right)$<br>Where $\mathrm{x}'$is the number of polymer segments. For a polymer that is not close to any polymers, $\mathrm{x}' = \phi_{P,1}\delta\nu$.<br>When two polymer chains share the volume element, $\delta\nu$, $\mathrm{x}' = (\phi_{P,1} + \phi_{P,2})\delta\nu$.	Following the same reasoning with the appropriate simplifications for our infinitely long chain,<br>$\frac{U_{mix}}{Nk_BT} = \kappa\phi_P$<br>$\kappa = \frac{(z-z_p)}{k_BT}\left(\epsilon_{PS} - \frac{\epsilon_{SS}}{2}\right)$
The energy change in the volume $\delta\nu$, when two chains occupy their own space and then a shared volume element then equals,<br>$\delta\left(\frac{U_{mix}}{Nk_B}\right) = -2\kappa\frac{\delta\nu}{V_S}\phi_{P,1}\phi_{P,2}$	For an infinitely long chain with periodic boundary conditions, multiple chains will not interact, so that we have,<br>$\delta\left(\frac{U_{mix}}{Nk_B}\right) = 0$

1.1.6 Entropic Contribution

Original FH Theory	Isolated Infinite Chain
We first follow the original derivation of Flory-Huggins theory as shown in Section S1.1.3. It is known that this expression is inapplicable to dilute polymer solutions, but it was heuristically assumed by Flory and Krigbaum that the entropic contribution kept the same functional form, but with an entropic component for the penalty of being in proximity to the polyer.[4]	
For the sake of the derivation, Flory and Krigbaum imagined the entropic change with a set distance between two chains, thus removing the configurational entropy of the polymer.<br>$-\frac{S_{mix}}{Nk_B} = \phi_S \ln\phi_S - \left(\psi - \frac{1}{2}\right)\phi_S\phi_P$	Because our system is constrained to an infinitely long chain, we showed in Section S1.1.3 that there is no configurational entropy. Recall from section S1.1.2 that the number of polymer-solvent contacts for such a system is simply, $m_{PS} = (z - z_p)\phi_P$, implies,<br>$-\frac{S_{mix}}{Nk_B} = -\left(\psi - \frac{1}{2}\right)\phi_P$
With this thought process, a confounding effect would occur when two polymer chains were near enough to each other for their hydrated regions to interact. If this were to occur in a volume element $\delta\nu$, the entropy change with that interaction becomes,	The infinitely long chain system in a periodic box will not allow multiple polymer chains to be near each other in a volume element $\delta\nu$, the entropy change with that interaction reduces to the trivial expression,

$$\delta\left(-\frac{S_{mix}}{Nk_B}\right) = \frac{\delta v}{V_S}\Big\{\left(1-\rho_{P,1}V - \rho_{P,2}V\right)\log\left(1-\rho_{P,1}V-\rho_{P,2}V\right) - \left(1-\rho_{P,1}V\right)\log\left(1-\rho_{P,1}V\right) - \left(\psi-\frac{1}{2}\right) \times \left[\left(1-\rho_{P,1}V-\rho_{P,2}V\right)\left(\rho_{P,1}V + \rho_{P,2}V\right) - \left(1-\rho_{P,1}V\right)\rho_{P,1}V - \left(1-\rho_{P,2}V\right)\rho_{P,2}V\right]\Big\}$$ $$\delta\left(-\frac{S_{mix}}{Nk_B}\right) = 2\psi\frac{\delta v}{V_S}\phi_{P,1}\phi_{P,2}$$	$$\delta\left(-\frac{S_{mix}}{Nk_B}\right) = 0$$

S2. Hyperparameters for Alchemical Computations

Several hyperparameters may be used to determine the solvation free energy using alchemical methods. Equation S1 shows the soft Lennard Jones (LJ) equation where the value λ is varied from one to zero in a series of windows to take an interaction potential from full strength to turned off in a smooth, reversible manner. Hyperparameters from the equation include n and α_{LJ}, where the former will change the distribution of λ-windows from linear to a higher concentration of points near $\lambda = 0$ as n is increased, and the latter changes how aggressively the potential is softened as λ gets closer to zero.

$$U = \lambda^n 4\epsilon\left\{\frac{1}{\left[\alpha_{LJ}(1-\lambda)+\left(\frac{r}{\sigma}\right)^6\right]^2} - \frac{1}{\alpha_{LJ}(1-\lambda)+\left(\frac{r}{\sigma}\right)^6}\right\} \qquad \text{(S1)}$$

The last hyperparameter we considered was the number of λ-windows.

Because the simulations reported in the main text were computationally expensive, this exercise of determining hyperparameters was performed beforehand with a smaller system. One hundred solvent beads solvated a LJ dimer, providing an accessible simulation duration for the ninety-six simulations presented here for Multi-state Bennett Acceptance Ratio (MBAR). After weighing the influence of each hyperparameter, we decided that the following values would be best applied to our study: n = 3, α_{LJ} = 0.6, $N_{Windows}$= 25.

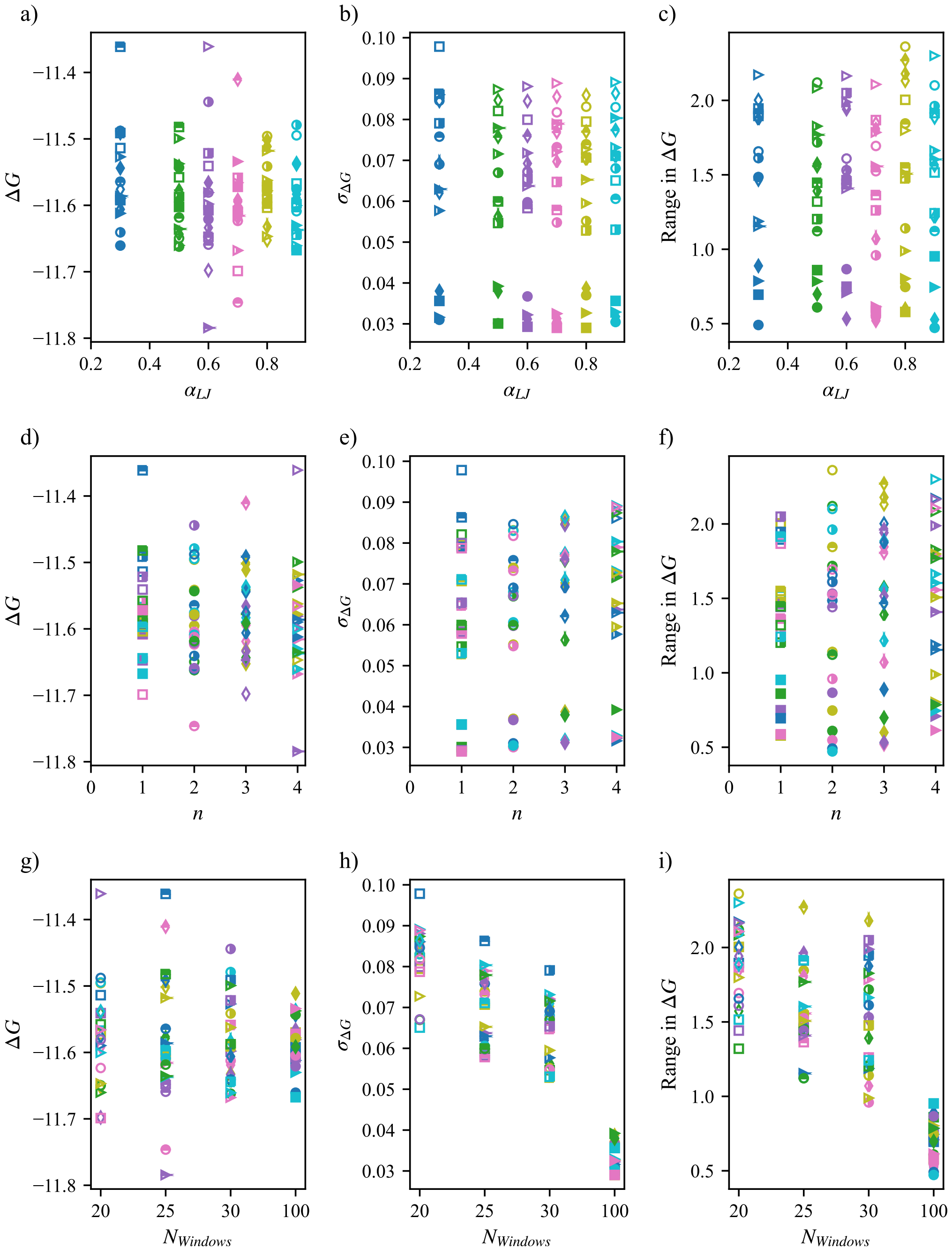

Figure S1: Comparison of alchemical hyperparameters for MBAR where the (a) free energy, ΔG, (b) the standard deviation in free energy, $\sigma_{\Delta G}$, and (c) the Range in ΔG when block averaging the free energy over the trajectory length plotted with respect to α, the parameter controlling how quickly the LJ potential "softens". These same properties are then plotted with respect to the LJ prefactor exponent n in λ^n: (d) ΔG, (e) $\sigma_{\Delta G}$, and (f) the Range in ΔG. The dependence of (g) ΔG, (h) $\sigma_{\Delta G}$, and (i) the Range in ΔG is plotted with respect to the number of windows, or steps taken for λ to change from a value of 1 to 0.

S3. Influence of Salt on Free Energy, Enthalpy, and Entropy Changes

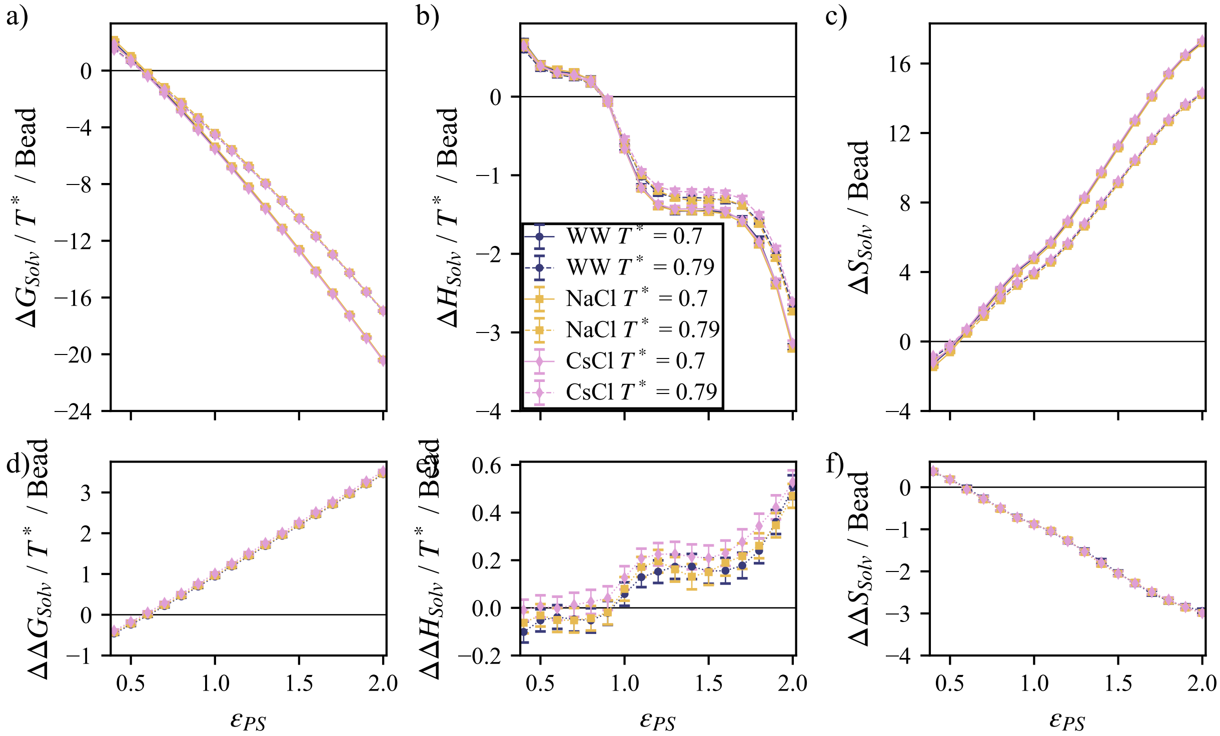

Figure S2: For a system without salt, the (a) free energy, (b) enthalpy, and (c) entropy of solvation, i.e., to decouple the polymer and solvent, are plotted with respect to interaction energy between polymer and solvent for two temperatures. The difference in solvation (d) free energy, (e) enthalpy, and (f) entropy between low and high temperatures illustrate temperature dependent solvation behavior. Uncertainty intervals are smaller than data markers and represent the standard deviation over three independent simulation boxes.

S4. Spherical Distribution Function

It is known that the Kirkwood-Buff integral (KBI) expression is difficult to evaluate because of the oscillations present in radial distribution functions (RDFs) over long length scales. Lockwood and Rossky used the observation that an RDF generated from an alternative choice in molecular center would yield the same KBI to present a method of generating a spherical distribution function as an average RDF over the alternative molecular centers in a sphere. Their work has since been applied to produce the spherical distribution with the expression:

$$\rho(\vec{r};\xi) = \frac{\int_{|\vec{r}'|<\xi} \rho(\vec{r}+\vec{r}')d\vec{r}'}{\frac{4}{3}\pi\xi^3} \qquad \text{[S2]}$$

where ξ is the spherical smoothing radius and ρ is the density of the system in that volume element. This expression has been used to determine the KBI of functional groups in a small solvent, but because our application is for isotropic single bead functional groups, we use the following single integral form:

$$\rho^{Sph}(r;\xi) = \frac{\int_{-\xi}^{\xi} \rho(r+r')\, P(r', r, \xi)\, dr'}{\frac{4}{3}\pi\xi^3} \qquad [S3]$$

Where $P(r', r, \xi)$ is a weighting function based on the surface area within the spherical smoothing radius that is at the distance, $r + r'$, corresponding to the value of $\rho(r + r')$. This surface area is not a straight plane, as illustrated in Figure S3 where the molecular center of a given RDF is taken at point *A*, and that a value of that RDF as point, *r* is smoothed by the RDF values contained in a sphere of radius, *r*', found around that point. Thus, the bounds of integration span from -ξ to ξ, plus or minus the smearing length, and each value is weighted by the surface area of the spherical cap of the sphere with a radius of *r*+*r*' within the sphere of the radius ξ.

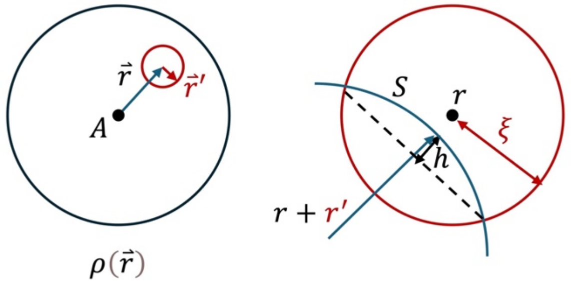

Figure S3: Illustration of surface area in generating the surface area expression used to weight a value in the radial distribution function according to the spherical smoothing function.

The surface area of the cap of a sphere is expressed as 2π times the radius of the sphere (in our case *r*+*r*') times the height of the sectioning plane.

$$P(r', r, \xi) = S = 2\pi(r + r')h \qquad [S4]$$

The value of h is taken from expressions for the intersection of two spheres. The height of the cap is then the radius of the sphere less the distance to the sectioning plane, *x*:

$$h = (r + r') - x = (r + r') - \frac{r^2 - \xi^2 + (r + r')^2}{2r} = \frac{\xi^2 - r'^2}{2r} \qquad [S5]$$

Thus the weighting function taken from the surface area of intersection is:

$$P(r', r, \xi) = \pi\frac{r + r'}{r}(\xi^2 - r'^2) \qquad [S6]$$

A test of the validity of this equation may be demonstrated through the exercise of showing that:

$$V = \int_{-\xi}^{\xi} P(r', r, \xi) dr' = 4\pi\xi^3/3 \qquad [S7]$$

Our expression for the spherical distribution function is then:

$$\rho^{Sph}(r;\xi) = \frac{3r}{4\xi^3} \int_{-\xi}^{\xi} \rho(r + r')\ \ (r + r')\left(\xi^2 - {r'}^2\right) dr' \qquad \text{[S8]}$$

This expression can be evaluated numerically in one dimension.

S5. Caging Relaxation Time with Respect to Temperature

The caging timescale t_c for the Debye-Waller parameter $\langle u^2 \rangle$ is taken to be the minimum of the derivative in the log-log plot of the mean-squared displacement (MSD).[5–7] Here we show the comparison for pure solvent at the temperature run in a previous work, $T^* = 0.75$,[8] and the two temperatures studied in this work, $T^* = 0.7$ and 0.79. The characteristic caging timescale was taken to be 0.8 τ previously, and with our results showing subtle variation with a change in temperature, we retain this characteristic time for the computation of the Debye-Waller parameter in this work.

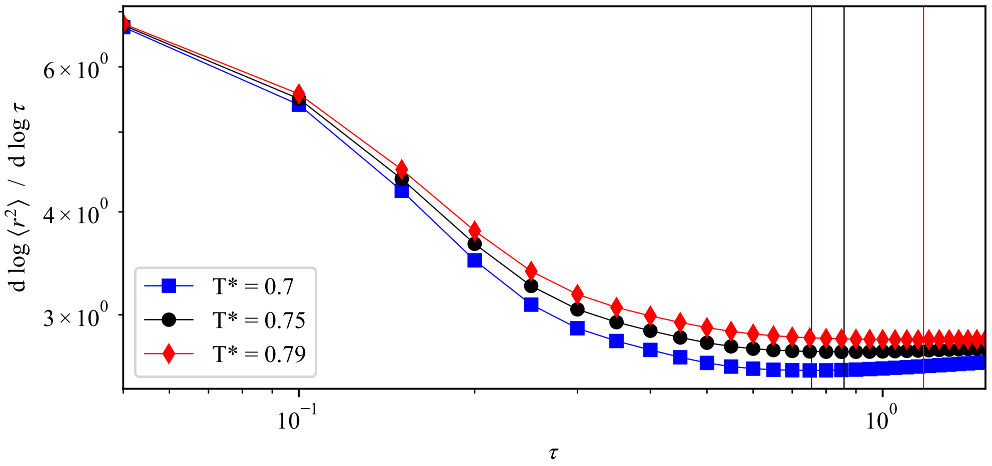

Figure S4: The derivative of the MSD log-log plot reveals the characteristic relaxation time on the caging timescale.

Temperature, T^* [ϵ]	t_C [τ]
0.7	0.76 ± 0.001
0.75	0.86 ± 0.01
0.79	1.17 ± 0.01

S6. CDF and Debye-Waller Profiles for Simulated Systems

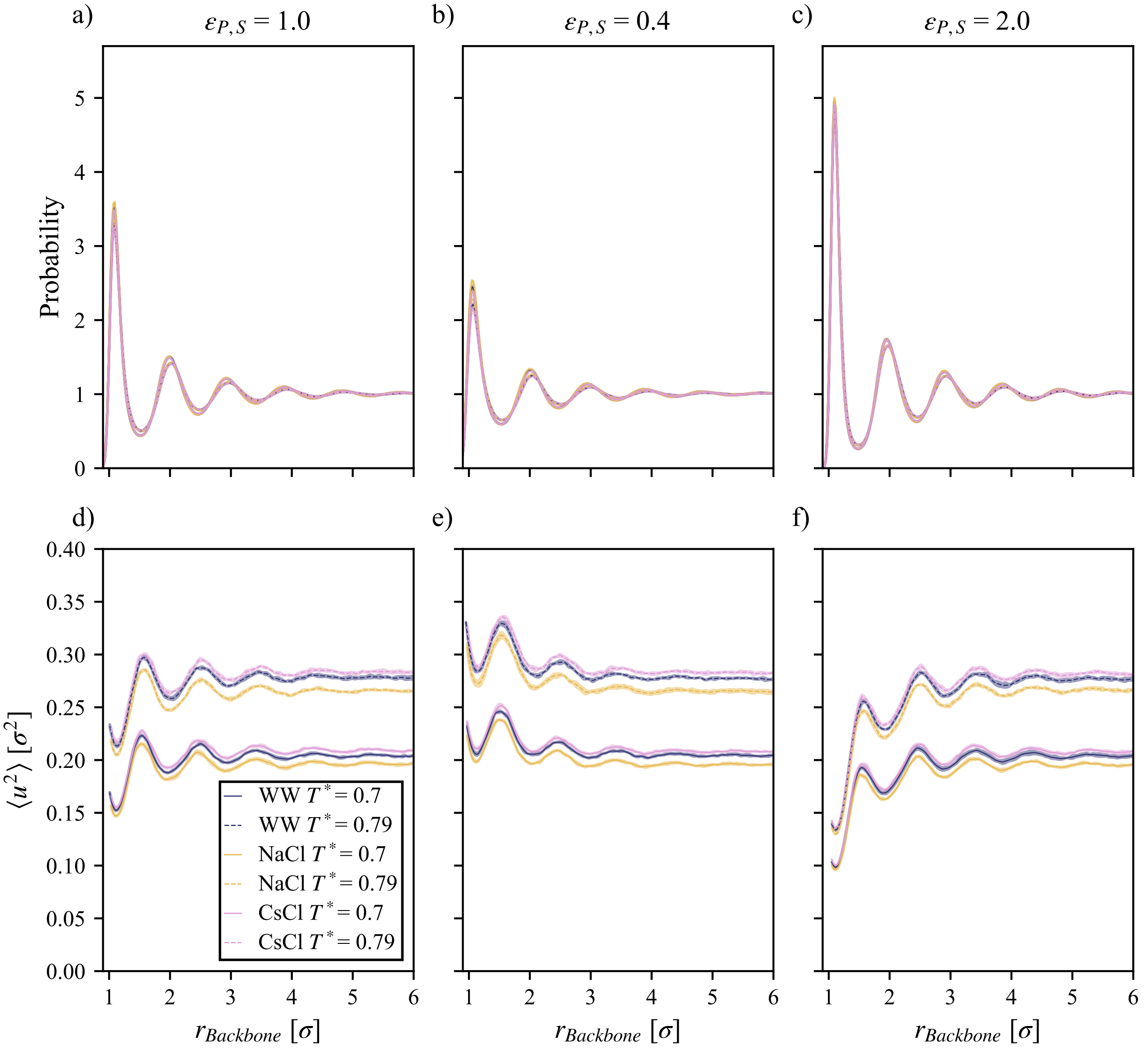

Figure S5: Cylindrical distribution function (CDF) between the polymer and solution (solvent and ions) of the varying polymer-solution interaction parameters (a) $\epsilon_{PS} = 0.4$, (b) $\epsilon_{PS} = 1.0$, and (c) $\epsilon_{PS} = 2.0$. The unscaled values of the Debye-Waller parameter with respect to distance from the polymer for varying polymer-solution interaction parameters (d) $\epsilon_{PS} = 0.4$, (e) $\epsilon_{PS} = 1.0$, and (f) $\epsilon_{PS} = 2.0$. Shaded regions represent the standard deviation over the three independent boxes, which may be smaller than the plotted trendline.

References

(1) Flory, P. J. Thermodynamics of High Polymer Solutions. *J. Chem. Phys.* **1942**, *10* (1), 51–61. https://doi.org/10.1063/1.1723621.

(2) Flory, P. J. *Principles of Polymer Chemistry*; The George Fisher Baker non-resident lectureship in chemistry at Cornell University; Cornell University Press: Ithaca, 1953.

(3) Huggins, M. L. Some Properties of Solutions of Long-Chain Compounds. *J. Phys. Chem.* **1942**, *46* (1), 151–158. https://doi.org/10.1021/j150415a018.

(4) Flory, P. J.; Krigbaum, W. R. Statistical Mechanics of Dilute Polymer Solutions. II. *J. Chem. Phys.* **1950**, *18* (8), 1086–1094. https://doi.org/10.1063/1.1747866.

(5) Starr, F. W.; Douglas, J. F.; Sastry, S. The Relationship of Dynamical Heterogeneity to the Adam-Gibbs and Random First-Order Transition Theories of Glass Formation. *J. Chem. Phys.* **2013**, *138* (12), 12A541. https://doi.org/10.1063/1.4790138.

(6) Pazmiño Betancourt, B. A.; Hanakata, P. Z.; Starr, F. W.; Douglas, J. F. Quantitative Relations between Cooperative Motion, Emergent Elasticity, and Free Volume in Model Glass-Forming Polymer Materials. *Proc. Natl. Acad. Sci.* **2015**, *112* (10), 2966–2971. https://doi.org/10.1073/pnas.1418654112.

(7) Pazmiño Betancourt, B. A.; Starr, F. W.; Douglas, J. F. String-like Collective Motion in the α - and β - Relaxation of a Coarse-Grained Polymer Melt. *J. Chem. Phys.* **2018**, *148* (10), 104508. https://doi.org/10.1063/1.5009442.

(8) Clark, J. A.; Douglas, J. F. Do Specific Ion Effects on Collective Relaxation Arise from Perturbation of Hydrogen-Bonding Network Structure? *J. Phys. Chem. B* **2024**, *128* (26), 6362–6375. https://doi.org/10.1021/acs.jpcb.4c02638.
